# Subtyping of Swine Influenza Viruses Using a High-Throughput Real-Time PCR Platform

**DOI:** 10.3389/fcimb.2018.00165

**Published:** 2018-05-22

**Authors:** Nicole B. Goecke, Jesper S. Krog, Charlotte K. Hjulsager, Kerstin Skovgaard, Timm C. Harder, Solvej Ø. Breum, Lars E. Larsen

**Affiliations:** ^1^Division for Diagnostics & Scientific Advice, National Veterinary Institute, Technical University of Denmark, Kongens Lyngby, Denmark; ^2^Institute of Diagnostic Virology, Federal Research Institute for Animal Health, Friedrich-Loeffler Institute, Riems, Germany

**Keywords:** swine influenza virus, subtyping, surveillance, real-time PCR, high-throughput real-time PCR, diagnostics

## Abstract

Influenza A viruses (IAVs) are important human and animal pathogens with high impact on human and animal health. In Denmark, a passive surveillance program for IAV in pigs has been performed since 2011, where screening tests and subsequent subtyping are performed by reverse transcription quantitative real-time PCR (RT-qPCR). A disadvantage of the current subtyping system is that several assays are needed to cover the wide range of circulating subtypes, which makes the system expensive and time-consuming. Therefore, the aim of the present study was to develop a high-throughput method, which could improve surveillance of swine influenza viruses (swIAVs) and lower the costs of virus subtyping. Twelve qPCR assays specific for various hemagglutinin and neuraminidase gene lineages relevant for swIAV and six assays specific for the internal genes of IAV were developed and optimized for the high-throughput qPCR platform BioMark (Fluidigm). The qPCR assays were validated and optimized to run under the same reaction conditions using a 48.48 dynamic array (48.48DA). The sensitivity and specificity was assessed by testing virus isolates and field samples with known subtypes. The results revealed a performance of the swIAV 48.48DA similar to conventional real-time analysis, and furthermore, the specificity of swIAV 48.48DA was very high and without cross reactions between the assays. This high-throughput system provides a cost-effective alternative for subtyping of swIAVs.

## Introduction

Swine influenza is a respiratory disease caused by multiple subtypes of influenza A virus (IAV). The genome of IAV consists of eight segments, which code for different virus proteins. Subtype classification of IAV is based on the encoded surface glycoproteins hemagglutinin (HA) and neuraminidase (NA), and so far, 16 different HA and nine different NA subtypes have been described together with two recently discovered bat-derived subtypes, H17N10 and H18N11 (Cheung and Poon, 2007; Wu et al., 2014). Influenza A virus contains further six “internal” gene segments which encode basic polymerase 2 (PB2), basic polymerase 1 (PB1), acidic polymerase (PA), nucleoprotein (NP), matrix (M1, M2), and non-structural proteins (NS1, NS2). These segments and their translation products have an essential role in the virulence and host specificity of a given IAV and can also impact the risk of transmission to humans (Bi et al., 2015).

The predominant swine IAV (swIAV) subtypes globally are H1N1, H3N2, and H1N2, which all show considerable diversity. The genetic and antigenic characteristics of IAVs in pigs differ depending on their geographic locations (Kuntz-Simon and Madec, 2009; Simon et al., 2014). In Europe, the dominant H1N1 swIAV is of avian origin, referred to as avian-like swine H1N1 (H1_av_N1_av_), which was introduced from waterfowl to pigs in the late 1970s (Pensaert et al., 1981; Simon et al., 2014). The dominant genotype of H3N2 virus in European pigs is the H3N2 (H3_sw_N2_sw_) virus that was introduced in 1984. The HA and NA genes of the H3_sw_N2_sw_ are of human origin, while the other six gene segments are of avian (H1_av_N1_av_) descent (Castrucci et al., 1993). In 1994, an H1N2 reassortant was isolated for the first time in United Kingdom and has subsequently been detected in many European countries. This human-like reassortant swine H1N2 (H1_hu_N2_sw_) virus comprised the HA gene from a human seasonal H1N1 virus, the NA gene from the H3_sw_N2_sw_ virus and internal genes from the H1_av_N1_av_ virus (Alexander et al., 1998). The dominating European H1_hu_N2_sw_ virus has never been detected in Denmark, however, a new reassortant H1_av_N2_sw_, containing the HA gene from the H1_av_N1_av_ virus and the NA gene from H3_sw_N2_sw_, was found in Denmark in 2003 (Trebbien et al., 2013). This avian-like H1N2 (H1_av_N2_sw_) virus has become established in Denmark and other European countries (Trebbien et al., 2013; Simon et al., 2014) and is now the most prevalent subtype circulating in Danish pigs. In 2009, a new human pandemic strain [A(H1N1)pdm09] entered the global swine population and is now enzootic in swine globally. Furthermore, an increasing number of reassortants between the predominant enzootic swIAVs and the A(H1N1)pdm09 virus have been observed, making subtyping of swIAV a very complex task (Starick et al., 2011; Watson et al., 2015). Furthermore, spillover of seasonal human H3 (H3_hu_) segments and human N2 (N2_hu_) have been observed in Danish swine (Breum et al., 2013; Krog et al., 2017).

In Denmark, a passive surveillance program for swIAVs has been conducted since 2011. A requirement for efficient swIAV surveillance is highly sensitive and specific diagnostic tests. Today, the swIAV screening test and subsequent subtyping is performed by reverse transcription (RT) quantitative real-time PCR (qPCR), where several different assays are needed to cover the wide range of circulating subtypes, which make detection and subtyping costly and time consuming. The aim of the present study was to establish a high-throughput method for detection and subtyping of swIAVs in Danish pigs. The BioMark dynamic array (DA) (Fluidigm, South San Francisco, USA) is capable of performing parallel qPCRs by combining e.g., 48 samples with 48 assays or 96 samples with 96 assays in a combinatorial manner inside the integrated fluidic circuit (IFC) resulting in either 2,304 or 9,216 individual reactions in a single run. Besides being able to process a high number of reactions in a single run, the high-throughput qPCR BioMark system also uses less sample and reagent volume compared to standard qPCR platforms (Spurgeon et al., 2008). The present study describes the design, optimization and validation of a swIAV 48.48DA; a setup consisting of 18 qPCR assays targeting the different swIAVs circulating in Europe.

## Materials and methods

### Samples

In the routine veterinary diagnostic laboratory at the National Veterinary Institute in Denmark, oral fluid, lung tissue, and nasal swabs are tested for swIAV from pigs with a history of respiratory disease. The samples are tested by an in-house modified RT-qPCR assay detecting the M gene (Trebbien et al., 2013). For selected swIAV positive samples, virus is isolated in Madin-Darby Canine Kidney (MDCK) cell cultures, followed by full genome sequencing by Next Generation Sequencing (NGS) (Krog et al., 2017). For validation of the swIAV 48.48DA a total of 32 field samples from 2015 and 2016 (Table 1) and 29 virus isolates for which full genome sequences were available were used (Table 2).

**Table 1:** HA and NA subtyping of swIAV from Danish field samples by swIAV 48.48DA.

**Sample name**	**Origin^a^**	**Subtype^b^**		**Singleplex^c^**	**swIAV 48.48DA^d^**										
		**HA**	**NA**	**M**	**M**	**H1_av_**	**H1_hu_**	**H1_pdm_**	**H3**	**N1_B1_**	**N1_B2_**	**N1_pdm_**	**N2_B1_**	**N2_B2_**	**N2_hu_**
**A/Swine/Denmark/7961-7/2016**	?	H1pdm	N1pdm	21	22.41			27.18		27.87	21.09	**24.52**	**30.66**		
A/Swine/Denmark/9186-1/2016	ns	H1pdm	N1pdm	29.57	28.73			22.53		25.84	29.97	29.45			
A/Swine/Denmark/10130-1/2016	ns	H1pdm	N1pdm	22	22.53			19.95		21.58	22.25	21.43			
A/Swine/Denmark/16219-1/2016	?	H1pdm	N1pdm	23	25.90			23.57		25.93	26.49	26.92			
A/Swine/Denmark/16966-2/2016	?	H1pdm	N1pdm	25.85	26.34			24.11		24.96	25.46	26,99			
A/Swine/Denmark/19295-1/2015	lu	H1pdm	N1pdm	21.50	21.43			15.93		18.76	19.80	19.53			
A/Swine/Denmark/19089-3/2015	lu	H1pdm	N1pdm	21	22.24			18.97		19.27	20.27	21.66			
A/Swine/Denmark/19090-1/2015	lu	H1pdm	N1pdm	24	25.58			20.05		25.63		23.61			
A/Swine/Denmark/20835-1/2015	?	H1pdm	N1pdm	24	23.78			23.83		21.52	22.32	22.18			
A/Swine/Denmark/6521-1/2016	ns	H1pdm	N2sw	27	24.99			26.42					28.24	32.63	
**A/Swine/Denmark/7988-2/2016**	lu	H1pdm	N2sw	18	18.50	**27.74**		**22.10**					24.92	25.79	
A/Swine/Denmark/9154-4/2016	ns	H1pdm	N2sw	26.44	26.18			23.28					27.83	32.56	
A/Swine/Denmark/20566-1/2015	ns	H1pdm	N2sw	19	19.10			14.89					26.71	31.26	
A/Swine/Denmark/10856-3/2016	ns	H1av	N1	26	25.72	31.59				29.11					
A/Swine/Denmark/23293-4/2015	ns	H1av	N1	25	24.47	32.85				25.02	26.57				
**A/Swine/Denmark/8938-1/2015**	ns	H1av	N? 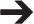 N1	31	25.53	23.91					24.95				
A/Swine/Denmark/6392-2/2016	ns	H1av	N2sw	21.39	23.45	22.64							26.43		
A/Swine/Denmark/6469-1/2016	lu	H1av	N2sw	20	21.94	28.10							27.24		
A/Swine/Denmark/6534-3/2016	lu	H1av	N2sw	15.87	18.78	18.18							22.06		
**A/Swine/Denmark/6598-1/2016**	sa	H1av	N2sw	29	26.30	28.91									
A/Swine/Denmark/6637-1/2016	ns	H1av	N2sw	25	25.68	25.54							25.26		
**A/Swine/Denmark/6686-1/2015**	ns	H? 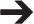 H1av	N2sw	30	25.25	24.09								34	
A/Swine/Denmark/8065-1/2016	ns	H1av	N2sw	22	23.62	24.37								28.77	
A/Swine/Denmark/9051-1/2016	lu	H1av	N2sw	18.75	20.92	19.80							25.17	29.47	
A/Swine/Denmark/9846-1/2016	ns	H1av	N2sw	25.84	25.35	25.24							29.89		
A/Swine/Denmark/11013-3/2016	lu	H1av	N2sw	14	14.34	16.52							17.67	20.67	
**A/Swine/Denmark/14170-2/2016**	?	H1av	N? 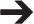 N2sw	25.41	23.51	23.95							27.94	31.68	
A/Swine/Denmark/15963-2/2016	?	H1av	N2sw	23	22.49	20.97							30.70	28.21	
A/Swine/Denmark/19292-1/2015	ns	H1av	N2sw	26.45	25.67	23.78							30.37	31.93	
A/Swine/Denmark/19293-1/2015	lu	H1av	N2sw	16.27	17.24	15.38							21.31	25.04	
A/Swine/Denmark/23653-3/2015	ns	H1av	N2sw	27.49	25.35	24.25							30.94	33.87	
**A/Swine/Denmark/9079-2/2016**	lu	H?	N2sw	27.31	26.39								37.51		

a *ns, nasal swab; lu, lung tissue; sa, oral fluid; ?, unknown*.

b *Subtype achieved by an in house multiplex RT-qPCR (modified from Henritzi et al., 2016)*.

c *Cq-value achieved by an in-house modified RT-qPCR assay detecting the M gene (Trebbien et al., 2013)*.

d *Cq-value achieved by swIAV 48.48DA*.

*Samples where a discrepancy was observed between the two analyses are in bold letter*.

**Table 2:**
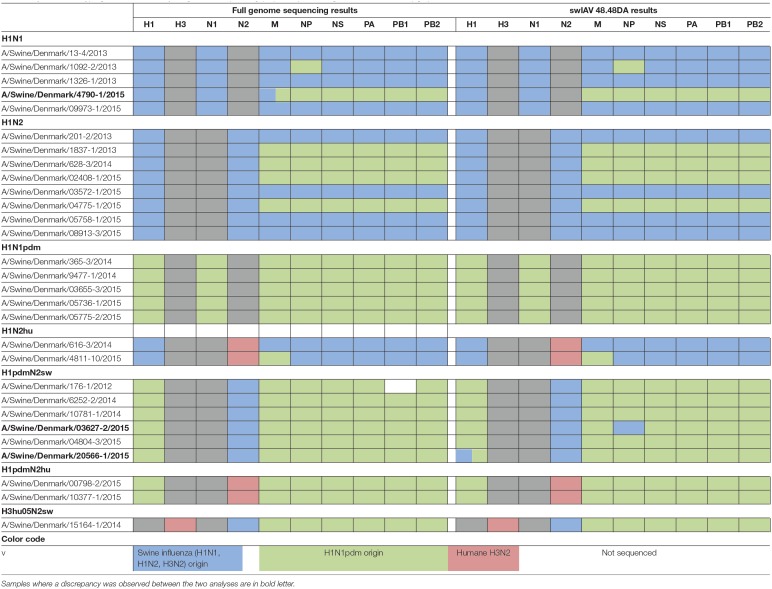
Parallel subtyping of virus isolates by full genome sequencing (left) and qPCR using the swIAV 48.48DA (right).

*Samples where a discrepancy was observed between the two analyses are in bold letter*.

### Primer and probe design

The swIAV 48.48DA was designed to include qPCR assays targeting the different lineages of H1, H3, N1, and N2 circulating in pigs in Europe. For the H1 subtypes the design aimed at differentiating between the H1 lineages; H1_av_, H1 from A(H1N1)pdm09 (H1_pdm_) and H1_hu_. For the H3 lineages the aim was to differentiate between H3_sw_ and H3_hu_. For the NA subtypes N1 and N2 broadly reacting assays (N1_B1_, N1_B2_, N2_B1_, N2_B2_) were included together with an assay specifically detecting the A(H1N1)pdm09 lineage of N1 (N1_pdm_) and an assay specifically detecting N2_hu_ derived from the seasonal human H3N2, that circulated in humans in the mid-1990s. Accordingly, N1_pdm_ positive viruses gave positive results with the N1_B1_, N1_B2_ and N1_pdm_ assays, while N2_hu_ positive viruses gave positive results with the N2_B1_, N2_B2_ and N2_hu_ assays.

In addition, six qPCR assays specific for the internal genes of A(H1N1)pdm09 (PB1_pdm_, PB2_pdm_, PA_pdm_, NP_pdm_, M_pdm_, NS_pdm_) were included. Primers and probes were either selected from previously published methods or designed in the present study. The final sets of primers and probes consisting of 18 PCR assays, of which 12 were designed *de novo*, two were from published literature, three were modified published assays and one was an in-house assay. The modifications are highlighted in bold in Tables 3, 4. New primer and probe sequences were designed based on alignments comprising full-length sequences of the eight gene segments from European swIAVs. The sequences were retrieved from Influenza Research Database^1^ The specificity of primers and probes was tested *in silico* by using BLAST search (Altschul et al., 1990), while melting temperature of the oligonucleotides was approximated using the online tool “OligoCalc” (Kibbe, 2007). The RT-qPCR assays were tested on the Rotor-Gene Q qPCR system (QIAGEN, Hilden, Germany) using a panel of six strains of cultured viruses, representing targets for one or more of the different primer and probe sets. RT-qPCR assays were performed in a final volume of 25 μL using QIAGEN OneStep RT-PCR kit (QIAGEN), with 5 μL of 5X QIAGEN One step RT-PCR buffer, 1 μL of 10 mM nucleotides dNTP mix, 1.25 μL of 25 mM MgCl_2_, 1 μL of 100 μM primers, 0.25 μL of 30 μM probe, 1 μL QIAGEN enzyme mix, 2 μL RNA and 12.5 μL RNase-free water. Thermal cycling conditions were as follow: 50°C for 30 min, 95°C for 15 min followed by 40 cycles at 94°C for 10 s, 54°C for 30 s and 72°C for 10 s. The fluorescence signal was acquired at the 54°C step in the Green channel (470–510 nm). Data was analyzed with the Rotor-Gene Q Series Software 2.3.1. (QIAGEN) with the following parameter adjustments: dynamic tube normalization, on; noise slope correction, on; ignore first cycle; outlier removal, 10%; quantification cycle (Cq) threshold fixed, 0.01. All reactions were run in duplicates and non-template control (nuclease-free water) was included in each run.

**Table 3:** Primers and probes for detection of M, HA, and NA genes.

**Primer/probe**	**Sequence (5′-3′)**	**Product size (bp)**	**References**
**H1**_av_			Modified from (Henritzi et al., 2016)^a^
H1_av_-F	GAAGGRGGATGGACAGG**A**AT**G**A	139	
H1_av_-R	CAATTAHTGARTTCACTTTGTTG**CTG**		
H1_av_-P	**FAM**-TCTGGTTACGCAGCWGATCAGAAAA-BHQ1		
**H1**_hu_		169	Primer: This study
H1_hu_-F	GGWTGGTATGGTTATCATCAT		Probe: (Bonin et al., 2018)
H1_hu_-R	CTCGATTACAGAGTTCACC		
H1_hu_-P	FAM-CAGGGATCTGGCTATGCTGCAGAYC-BHQ1		
**H1**_pdm_		87	In-house assay
H1_pdm_-F	AGTTCAAGCCGGAAATAGCA		
H1_pdm_-R	CCCGGCTCTACTAGTGTCCA		
H1_pdm_-P	FAM-CCCAAAGTGAGGRATCAAGAAGGGAG-BHQ1		
**H3**_hu_		93	This study
H3_hu_-F	TGATGGAGAAAACTGCACACTA		
H3_hu_-R	CGTTCAACAAAAAGGTCCCATTTC		
H3_hu_-P	FAM-CACACTGAGGGTCTCCCAATAGAGCATCTA-BHQ1		
**H3**_sw_		93	This study
H3_sw_-F	TGATGGAGCAAATTGCACACTG		
H3_sw_-R	CGTTCAATGAAAAGGTCCCATTTC		
H3_sw_-P	FAM-CACAATGAGGGTCCCCTAATAGAGCGTCCA-BHQ1		
**N1**_B1_		99	This study
N1_B1_-F	CCTTGCTTCTGGGTTGAACTAATC		
N1_B1_-R	AGTGTCACTATTTACACCACAAAAGG		
N1_B1_-P	FAM-TGCTCCCGCTAGTCCAGATTGTGTTCTCTT-BHQ1		
**N1**_B2_		126	Henritzi et al., 2016
N1_B2_-F	AGRCCTTGYTTCTGGGTTGA		
N1_B2_-R	ACCGTCTGGCCAAGACCA		
N1_B2_-P	FAM-ATYTGGACYAGTGGGAGCAGCAT-BHQ1		
**N1**_pdm_		102	This study
N1_pdm_-F	CGAAATGAGTGCCCCTAATTATC		
N1_pdm_-R	CGATTCGAGCCATGCCAGTTA		
N1_pdm_-P^*^	FAM-[+C][+C]T[+G]ATTCT[+A]GTGAAATCA[+C]-BHQ1		
**N2**_B1_		101	This study
N2_B1_-F	TATTGATGAATGAGTTGGGTGTTCC		
N2_B1_-R	ATGCAGCCATGCTTTTCCATC		
N2_B1_-P	FAM-TGAACTGGACCATGCTATACACACTTGCCT-BHQ1		
**N2**_B2_		116	Modified from (Henritzi et al., 2016)^a^
N2_B2_-F	AGTCTGGTGGACYTCAAAYAG		
N2_B2_-R	TTGCGAAAGCTTATATAGVCATGA		
N2_B2_-P	**FAM**-CCATCAGGCCATGAGCCTGWWCCATA-BHQ1		
**N2**_hu_		92	This study
N2_hu_-F	CTGGTATTTTCTCTGTTGAAGGC		
N2_hu_-R	CCASACTTCAKTTTCCTGYTTCC		
N2_hu_-P^*^	VIC-T[+C]A[+A]CTCYACATAAAAGCACC[+G]-BHQ1		
**M**		204	(Loeffen et al., 2011)
M-F	CTTCTAACCGAGGTCGAAACGTA		
M-R	CACTGGGCACGGTGAGC		
M-P	FAM-TCAGGCCCCCTCAAAGCCGA-BHQ1		

a *Letters in bold in the sequences indicate the modification compared to the published sequences*.

* *Locked Nucleic Acid positions are indicated in brackets*.

**Table 4:** Primers and probes for detection of the internal pandemic genes of swIAVs.

**Primer/probe**	**Sequence (5′-3′)**	**Product size (bp)**	**References**
**PB2**_pdm_			
PB2_pdm_-F	GATAGTAAGCGGGAGAGAC	128	This study
PB2_pdm_-R	GCTGGTTTGCCCTATTGAC		
PB2_pdm_-P	FAM-GCTGAGGCAATAATTGTGGCCATGG-BHQ1		
**PB1**_pdm_			
PB1_pdm_-F	CAAAGACTACAGATACACATATAG	124	This study
PB1_pdm_-R	ATCTGATACTAATAGCCCTAC		
PB1_pdm_-P	FAM-GGGGAGACACACAAATTCAGACGAG-BHQ1		
**PA**_pdm_			
PA_pdm_-F	GGTGAAAATATGGCACCAGAA	110	This study
PA_pdm_-R	TGCTAGAGATCTGGGCTC		
PA_pdm_-P	FAM-GTAGACTTTGATGAYTGCAAAGATGTTGG-BHQ1		
**NP**_pdm_			
NP_pdm_-F	ACGGTCAGCACTCATTCTG	117	This study
NP_pdm_-R	ACCAGTGAGTACCCTTCC		
NP_pdm_-P	FAM-TCATGCCCACTTGCTACTGCAAGC-BHQ1		
**M**_pdm_			
M_pdm_-F	CTGGCTAGCACTACRGCA	99	This study
M_pdm_-R	TACCATYTGCCTAGTCTGATTA		
M_pdm_-P	FAM-CTCYATGGCCTCTGCTGCCTGT-BHQ1		
**NS**_pdm_			
NS_pdm_-F	GAGGAAATGTCACGAGACTG	119	This study
NS_pdm_-R	ACTGAAGTTCGCTTTCAGTAC		
NS_pdm_-P	FAM-TTCCATGACCGCCTGGTCCAATCG-BHQ1		

Primers and the dual labeled probes were purchased from Eurofins Genomics (Ebersberg, Germany), while Locked Nucleic Acid (LNA) probes were from BioNordika (Herlev, Denmark). Primers and probes were stored at −20°C.

### RNA extraction

Viral RNA was extracted from cultured viruses, oral fluid, lung tissue and nasal swab samples by RNeasy Mini Kit (QIAGEN) according to the manufacturer's instructions. Cell culture supernatant, oral fluid and nasal swab samples were prepared by mixing 200 μL material with 400 μL RLT buffer containing β-mercaptoethanol (Sigma-Aldrich, Brøndby, Denmark). Lung tissue samples were prepared by homogenization of 70 mg lung tissue in 1,400 μL RLT buffer containing β-mercaptoethanol (Sigma-Aldrich) on a TissueLyser II (QIAGEN) at 30 Hz in 3 min. The homogenate was centrifuged for 3 min at 12,000 g, and RNA was extracted from of 600 μL of the supernatant. Viral RNA was eluted in 60 μL RNase-free water and stored at −80°C.

### cDNA synthesis and pre-amplification

cDNA synthesis and pre-amplification of the extracted samples was performed in one step. Briefly, reaction volumes of 25 μL containing 1.50 μL of 10 μM random hexamer (Invitrogen, Carlsbad, California, USA), 0.75 μL primer mix (containing all qPCR primers (200 nM each) listed in Tables 3, 4), 5 μL of 5X QIAGEN One step RT-PCR buffer (QIAGEN), 1 μL of 10 mM nucleotides dNTP mix, 1.25 μL of 25 mM MgCl_2_, 1 μL QIAGEN enzyme mix, 3 μL sample and RNase-free water were prepared. cDNA synthesis and pre-amplification were performed on a T3 Thermocycler (Biometra, Fredensborg, Denmark) at 50°C for 30 min followed by enzyme inactivation at 95°C for 15 min followed by 24 cycles of 94°C for 10 s, 54°C for 30 s, and 72°C for 10 s. The pre-amplified cDNA was stored at −20°C.

### Preparation of the 48.48DA and qPCR

Pre-sample mix was prepared using the following components per sample; 3 μL TaqMan Gene Expression Master Mix (Applied Biosystems, Foster city, USA) and 0.3 μL 20x Sample loading reagent (Fluidigm, South San Francisco, USA). Pre-sample mix (3.3 μL) was mixed with 2.7 μL pre-amplified cDNA. Two different mixes of primers and probes with different concentrations, was prepared for each assay by mixing 3 μL primer/probe-stock (containing either 30 μM of each primer and 6.8 μM of probe or 33 μM of each primer and 10 μM of probe) with 3 μL 2X Assay loading reagent (Fluidigm). qPCR was performed in a BioMark 48.48DA (Fluidigm) combining 48 pre-amplified samples with 48 assays for 2304 individual and simultaneous qPCR reactions. The 48.48DA was primed in the IFC controller MX (Fluidigm) prior to loading of samples and assays. Sample mix (4.9 μL), and primer mix (4.9 μL) was dispensed into inlets on the 48.48DA, which was again placed in the IFC controller for loading and mixing of the 48 samples and 48 assays. After approximately 55 min the 48.48DA was ready for thermal cycling in the high-throughput qPCR instrument BioMark (Fluidigm) with the following cycling conditions: 15 min at 95°C, followed by 40 cycles at 94°C for 10 s, at 54°C for 30 s, and 72°C for 10 s. Non-template controls were included to control non-specific amplification and sample contamination. Specificity and sensitivity of all assays were tested against six virus isolates, representing targets for one or more of the different assays and thus the virus isolates functioned as both positive and negative controls for the individual primer and probe sets. Data (Cq-values and amplification curves) were acquired on the BioMark system and analyzed using the Fluidigm Real-Time PCR Analysis software 4.1.3 (Fluidigm).

### Validation of sensitivity of the qPCR assays

To test and compare the performance and dynamic range of the qPCR assays on the Rotor-Gene Q platform and on the high-throughput qPCR BioMark platform, RNA 10-fold serial dilutions from six different swIAV isolates were tested on the Rotor-Gene Q, and the same RNA dilutions were cDNA synthesized and pre-amplified and then tested on the BioMark. Furthermore, 10-fold serial dilutions were made from the pre-amplified cDNA from the six swIAV isolates and these were only tested on the BioMark platform.

### Verification of the specificity of the swIAV 48.48DA

The performance of the swIAV 48.48DA was verified by testing 32 field samples (nasal swabs, oral fluid, and lung tissue samples) and 29 virus isolates (Tables 1, 2). The full genome sequences were known for the virus isolates (Supplementary Table 1), while only the type of HA and NA genes were known for the field samples. The field samples have previously been tested and subtyped by an in-house multiplex RT-qPCR (modified from Henritzi et al., 2016) for diagnostic purposes.

### Data availability statement

The raw data supporting the conclusions of this manuscript will be made available by the authors, without undue reservation, to any qualified researcher.

## Results

### Specificity and sensitivity of the qPCR assays

**Figure 1:**
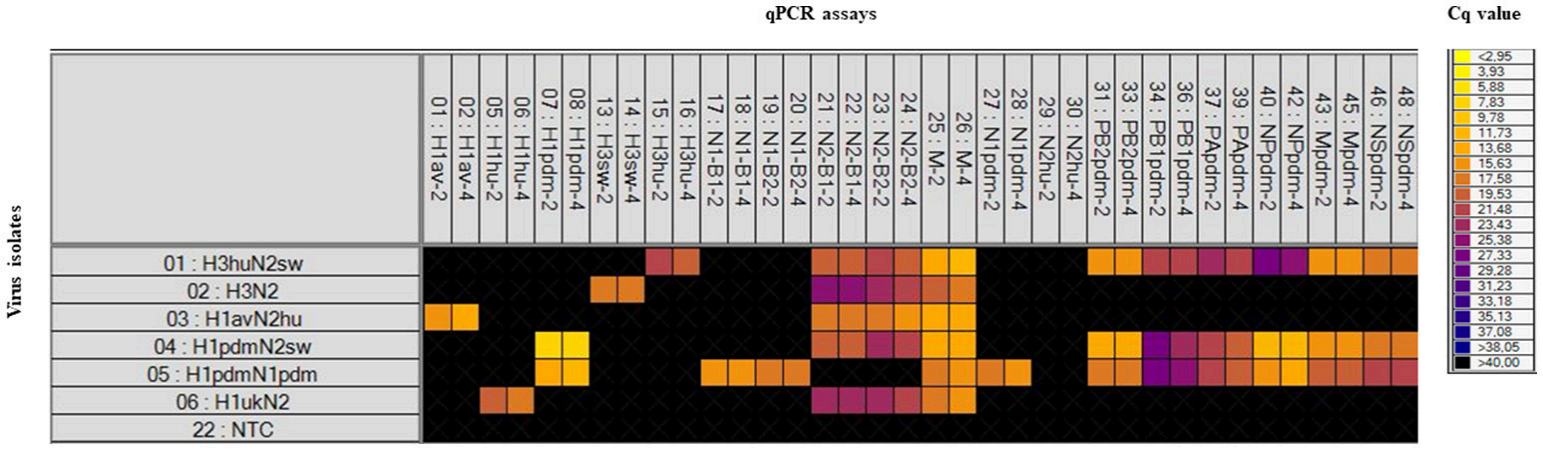
Heat map showing the specificity of the qPCR assays included on the swIAV 48.48DA by testing six virus isolates with known subtype (based on full genome sequencing). Top: The qPCR assays (Tables 3, 4) in two different primer/probe concentrations (indicated by the numbers two or four). Left: The virus isolates and a Non-Template Control (NTC). Each square corresponds to a single real-time PCR reaction. Cq-values for each reaction are indicated by color; the corresponding color scale is presented in the legend on the right. A black square is considered as a negative result.

RNA obtained from a panel of six IAVs of subtype H1, H3, N1, and N2 of avian, human, or porcine origin was used to evaluate the sensitivity and specificity of the different sets of primers and probes. The specificity of each assay was assessed from the Cq-value obtained from their respective target in relation to any cross reaction. For all qPCR assays, specific positive reactions were registered and no cross reactions were observed (Figure 1). The 18 selected assays discriminated correctly between the different linages of the HA gene (H1_av_, H1_hu_, H1_pdm_, H3_hu_, H3_sw_) and NA gene (N1_av_, N1_pdm_, N2_sw_, N2_hu_). The qPCR assays specific for the internal genes discriminated in all cases between the pandemic and non-pandemic genes (Figure 1). Series of 10-fold diluted RNA of the six virus isolates were tested on the Rotor-Gene Q and on the swIAV 48.48DA to assess the relative analytical sensitivity of the qPCR assays. Comparisons of the Cq-values of the dilutions revealed that, in general, the dynamic range of the assays was 2–5 log_10_ for the swIAV 48.48DA and four-six log_10_ for the Rotor-Gene system (Table 5). For some of the assays the undiluted sample was not tested due to too small amount of available sample material. The dynamic range of the qPCR assays was generally 1–2 log higher using the Rotor-Gene Q compared to the swIAV 48.48DA. Ten-fold serial dilutions of the pre-amplified cDNA resulted in similar dynamic range and efficiency as the RNA dilutions for each of the qPCR assays (data not shown).

**Table 5:** Relative sensitivity of qPCR assays on the Rotor-Gene Q platform and on the swIAV 48.48DA (BioMark platform).

	**Assays**											
**Dilution**	**H1_av_**		**H1_hu_**		**H1_pdm_**		**H3_hu_**		**H3_sw_**		**M**	
	**Rotor-Gene**	**BioMark**	**Rotor-Gene**	**BioMark**	**Rotor-Gene**	**BioMark**	**Rotor-Gene**	**BioMark**	**Rotor-Gene**	**BioMark**	**Rotor-Gene**	**BioMark**
10	14.20	17.29^*^	15.57	17.40	14.58	13.44	-	17.71	13.93	14.22	-	11.85
10^−1^	18.60	23.79	19.03	22.46	17.56	17.98	22.87	21.22	17.05	16.87	14.06	17.33
10^−2^	22.69	28.12	22.66	26.03	20.79	19.98	25.81	23.78	20.44	19.43	17.00	22.01
10^−3^	25.75	31.49	25.88	29.13	24.17	23.64	29.43	27.53	24.04	24.07	20.34	23.69
10^−4^	28.86	neg	29.30	neg	27.20	28.88	32.70	neg	28.16	28.27	24.09	27.81
10^−5^	32.66	neg	32.42	neg	30.97	neg	35.28	neg	neg	neg	27.53	29.45
10^−6^	neg	neg	neg	neg	34.22	neg	neg	neg	neg	neg	29.96	neg
10^−7^	neg	neg	neg	neg	neg	neg	neg	neg	neg	neg	neg	neg
Effectivity	0.89	0.80	0.98	0.80	1.01	0.88	1.07	1.04	0.91	1.01	1.02	0.84
**Dilution**	**N1_B1_**		**N1_B2_**		**N1_pdm_**		**N2_B1_**		**N2_B2_**		**N2_hu_**	
	**Rotor-Gene**	**BioMark**	**Rotor-Gene**	**BioMark**	**Rotor-Gene**	**BioMark**	**Rotor-Gene**	**BioMark**	**Rotor-Gene**	**BioMark**	**Rotor-Gene**	**BioMark**
10	18.09	15.15	19.14	17.21	18.04	16.24	14.54	15.53^*^	12.47	15.34	12.70	7.73
10^−1^	22.05	19.69	22.42	21.55	21.01	20.73	17.15	21.35	16.20	21.08	14.71	12.81
10^−2^	25.77	21.55	25.17	23.91	25.15	22.39	20.19	25.74	19.03	25.81	18.35	16.88
10^−3^	29.50	25.57	28.76	27.14	28.75	26.79	23.36	29.32	22.00	28.78	22.28	18.95
10^−4^	32.70	29.25	32.22	29.67	31.26	28.40	26.43	32.32	24.89	30.83	26.50	21.70
10^−5^	36.33	neg	35.76	neg	neg	neg	30.93	neg	29.31	neg	30.25	23.58
10^−6^	neg	neg	neg	neg	neg	neg	neg	neg	neg	neg	34.42	neg
10^−7^	neg	neg	neg	neg	neg	neg	neg	neg	neg	neg	neg	neg
Effectivity	0.89	1.01	1.00	1.02	0.96	1.07	1.04	0.89	1.04	0.82	0.89	1.11
**Dilution**	**PB2_pdm_**		**PB1_pdm_**		**PA_pdm_**		**NP_pdm_**		**NS_pdm_**		**M_pdm_**	
	**Rotor-Gene**	**BioMark**	**Rotor-Gene**	**BioMark**	**Rotor-Gene**	**BioMark**	**Rotor-Gene**	**BioMark**	**Rotor-Gene**	**BioMark**	**Rotor-Gene**	**BioMark**
10	15.32	13.79	-	19.67	-	18.49	-	-	-	16.45	13.81	12.58
10^−1^	20.13	16.78	22.08	22.91	17.88	21.76	18.22	17.25	17.26	20.35	16.82	17.14
10^−2^	25.27	19.31	24.62	25.85	22.12	24.67	21.59	20.97	20.30	23.26	20.62	20.16
10^−3^	27.6	22.35	27.74	29.02	24.94	28.27	24.88	25.09	23.80	26.06	24.46	24.36
10^−4^	29.95	26.87	31.53	32.14	28.19	32.56	29.11	27.68	27.16	28.18	27.93	28.07
10^−5^	neg	29.49	34.65	neg	32.61	neg	32.18	neg	30.64	neg	30.53	neg
10^−6^	neg	neg	37.43	neg	neg	neg	neg	neg	neg	neg	33.67	neg
10^−7^	neg	neg	neg	neg	neg	neg	neg	neg	neg	neg	neg	neg
Effectivity	0.87	1.00	1.07	1.09	0.93	0.94	0.92	0.89	0.98	1.06	0.98	0.82

### Validation of the swIAV 48.48DA chip

In order to validate the performance of the swIAV 48.48DA for subtyping of swIAVs, a total of 29 well-characterized virus isolates and 32 field samples were tested. The subtype of the samples had previously been determined by either full genome sequencing or multiplex RT-qPCR and the results obtained by the swIAV 48.48DA were compared to these findings (Tables 1, 2).

Of the 29 virus isolates, which have previously been full genome sequenced, 27 showed identical results when the subtyping was performed on the swIAV 48.48DA and by sequencing (Table 2). For each of the remaining two isolates there was a discrepancy for one of the genes. By full genome sequencing, the M gene of A/Swine/Denmark/4790-1/2015 had 93% identity with both pandemic and non-pandemic M genes of Danish swIAV strains (results not shown). The sample gave a positive signal for M_pdm_ on the swIAV 48.48DA despite that there were two mismatches in the primer and probe bindings regions and was by that defined as M_pdm_. The NP gene of A/Swine/Denmark/03627-2/2015 was subtyped as being of non-pandemic origin by the swIAV 48.48DA, while based on the full genome sequence analysis the NP gene was found to be pandemic. The sequence analysis also revealed one mismatch in the binding site of the reverse primer and two mismatches between the probe binding sites for the NP_pdm_ assay. Thus, these mutations could explain the discrepancy between the results obtained by sequencing and by test on the swIAV 48.48DA. Another sample, A/Swine/Denmark/ 20566-1/2015, was found to have the subtype H1_pdm_N2_sw_ with pandemic internal genes in both the full genome sequencing and when tested on the swIAV 48.48DA. However, this sample also tested positive in the assay specific for the H1_av_ gene (Table 2). Retesting of the sample on the Rotor-Gene Q in the H1_av_ and H1_pdm_ assays confirmed these results, indicating that this sample contained two different viruses.

**Figure 2:**
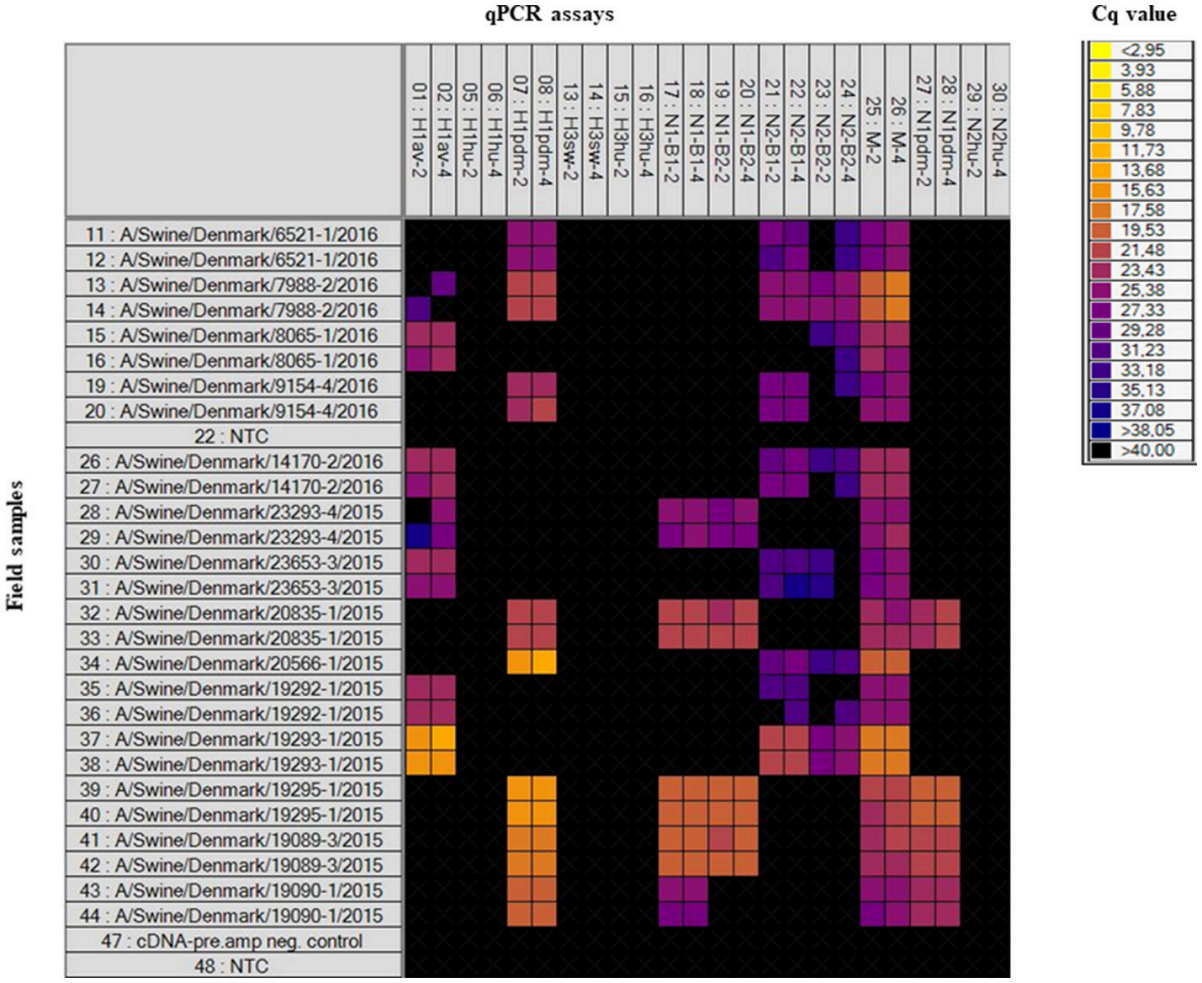
Heat map showing the results of a subset of the tested field samples on the swIAV 48.48DA. Top: The HA, NA, and M specific assays in two different primer/probe concentrations (indicated by the numbers two or four). Left: number 11–44 (except for number 22) is the tested field samples in duplicates, number 47 is a negative control for the cDNA-pre.amp setup and number 22 and 48 is Non-Template Controls (NTC). Each square corresponds to a single real-time PCR reaction. Cq-values for each reaction are indicated by color; the corresponding color scale is presented in the legend on the right. A black square is considered as a negative result.

Field samples (*n* = 32), consisting of nasal swabs, lung tissue or oral fluid, were also analyzed on the swIAV 48.48DA (Table 1). These samples had previously been subtyped by an in-house multiplex RT-qPCR assay (modified from Henritzi et al., 2016), thus only the HA and NA genes were known for these samples. The heat map in Figure 2 shows the results of a subset of the tested field samples in which the subtype for each of the sample was clarified based on the Cq-value and on the accuracy of the corresponding amplification curve. The swIAV 48.48DA and the multiplex RT-qPCR revealed the same HA type for 30 of the samples. However, none of the qPCR methods could define the HA subtype of sample A/Swine/Denmark/9079-2/2016. Furthermore, the HA subtype was not defined by the multiplex RT-qPCR for sample A/Swine/Denmark/6686-1/2015, but it was successfully determined using the swIAV 48.48DA. The sample A/Swine/Denmark/7988-2/2016 was found positive in both the H1_av_ and H1_pdm_ assay by the swIAV 48.48DA, but only positive for H1_pdm_ in the multiplex RT-qPCR. Therefore, this sample was further tested in the H1_av_ and H1_pdm_ assays on the Rotor-Gene Q, where it was found positive in both assays indicating infection with two different viruses. For the NA assays, 29 of 32 samples were found to have the same NA linage by both qPCR typing methods. For the sample A/Swine/Denmark/6598-1/2016 no signal was obtained in any of the NA assays on the swIAV 48.48DA, while it was positive in the N2_B2_ assay in the multiplex RT-qPCR. The sample was also tested in the N2 assay on the Rotor-Gene Q, where it was found to be weakly positive, with a Cq-value around 30. For the samples A/Swine/Denmark/14170-2/2016 and A/Swine/Denmark/8938-1/2015, no NA signal was obtained in the multiplex RT-qPCR, while the swIAV 48.48DA detected a signal in the N2_B1_ and N2_B2_ assays and in the N1_B2_, respectively. The swIAV 48.48DA found sample A/Swine/Denmark/7961-7/2016 to be of both N1_pdm_ and N2_sw_ origin, while this sample was only positive in the N1_pdm_ assay when using the multiplex RT-qPCR. Additional test on the Rotor-Gene Q found also this sample to be positive in the N2_B1_ assay—again indicating that the samples contained two different viruses.

In summary, when comparing the results for the swIAV 48.48DA with the sequencing and multiplex RT-qPCR results for the virus isolates and field samples, fully matching subtyping results (based on HA and NA genes) were obtained for 57 (29 virus isolates and 28 field samples) of 61 tested samples, and three of the 57 samples also showed an additional subtype in the analysis with the swIAV 48.48DA indicating a double infection. Furthermore, when comparing the number of positive findings in the gold-standard tests (sequencing and multiplex RT-qPCR) with the swIAV 48.48DA test an agreement was observed for nine of the tested genes, while a difference between 1.2 and 4.9 % was observed for the rest of the genes (Table 6).

**Table 6:** Comparison of the number of positive findings using the gold-standard test compared to the swIAV 48.48DA (BioMark) test (percentage in parentheses).

**Genes**	**Gold-standard test**	**swIAV 48.48DA (BioMark)**
H1_av_	32/61 (52.5%)	35/61 (57.4%)
H1_pdm_	26/61 (42.6%)	26/61 (42.6%)
H3_sw_	0/61 (0%)	0/61 (0%)
H3_hu_	1/61 (1.6%)	1/61 (1.6%)
N1	7/61 (11.5%)	8/61 (13.1%)
N1_pdm_	14/61 (23.0%)	14/61 (23.0%)
N2	34/61 (55.7%)	35/61 (57.4%)
N2_hu_	4/61 (6.6%)	4/61 (6.6%)
PB2_pdm_	19/29 (65.5%)	19/29 (65.5%)
PB1_pdm_	18/28 (64.3%)	19/29 (65.5%)
PA_pdm_	19/29 (65.5%)	19/29 (65.5%)
M_pdm_	20/29 (69.0%)	20/29 (69.0%)
NP_pdm_	20/29 (69.0%)	19/29 (65.5%)
NS_pdm_	19/29 (65.5%)	19/29 (65.5%)

## Discussion

The BioMark high-throughput qPCR protocol for detection and expanded subtyping of influenza virus in pigs described in the present paper proved to be as specific and sensitive as standard state-of the art diagnostic methods based on “conventional” qPCR and sequencing. This new approach makes it possible to combine multiple assays and samples and run them simultaneously. It requires less labor and pipetting, leading to an economical benefit. Another benefit is the use of nanolitre volume chambers in the DA, in contrast to conventional qPCR that uses microliter, thereby decreasing the use of expensive reagents. The BioMark high-throughput qPCR system has for years been widely used in research studies i.e., for the study of innate immune response to pathogens (Skovgaard et al., 2013). More recently, high-throughput qPCR protocols using the BioMark platform have also been designed as surveillance tools for tick-borne diseases and for food- and waterborne pathogens (Ishii et al., 2013; Michelet et al., 2014). Similar to the present study, Ishii et al. (2013) found the system to offer highly sensitive and specific simultaneous quantification of multiple food-and waterborne pathogens in multiple samples (Ishii et al., 2013). The platform is a flexible tool because it is easy to modify the assay panel by adding or removing primers or probes when new pathogens or new variants emerge (Ishii et al., 2013; Michelet et al., 2014).

To our knowledge this is the first paper describing the use of the BioMark high-throughput qPCR platform for detection and subtyping of influenza viruses. In general, there was a high degree of agreement for the results provided by multiplex RT-qPCR or sequencing and the results generated by the swIAV 48.48DA. For a few of the tested samples, there was a discrepancy. These differences could be explained by either co-infection with two viruses or by mismatches in the primer/probe binding regions. Thus, imperfect match between the target sequence and the primer and/or probe sequences can result in a false-negative signal even though the sample is positive for swIAV. This emphasizes that the swIAV 48.48DA or multiplex RT-qPCR protocols cannot stand alone as a subtyping method, but has to be combined with a continuous surveillance by sequencing of circulating swIAV isolates. Due to the high mutation- and reassortant rate of IAVs (Simon et al., 2014) it is important to do continuous sequencing of selected isolates because changes will occur over time and it is necessary to adjust the PCR assays accordingly. Sequencing is a very informative tool and it can contribute with indispensable information about evolutionary relationships based on similarities and differences between the sequences. However, since the number of isolates that can be sequenced is limited by practical and economic reasons, the swIAV 48.48DA provides an excellent screening tool for selection of atypical isolates for downstream characterization by sequencing.

Pre-amplification of the RNA samples was needed because of the very small sample volumes (< 10 nL; Korenková et al., 2015) in the reaction chambers. This is in accordance with recommendations from the supplier and previous studies using the BioMark protocols for the detection of i.e., water-borne pathogens (Ishii et al., 2013). The supplier of the BioMark platform recommends performing the cDNA synthesis and pre-amplification as two separate steps. However, we managed to change this into a one-step procedure by combining the cDNA synthesis and pre-amplification, which further reduced the analysis costs and the number of handling steps. A benefit of this alteration is also the reduced risk of contamination due the fewer handling steps. The swIAV 48.48DA was tested against a panel of representative virus isolates in order to assess the sensitivity and specificity. All the assays had an acceptable PCR efficiency between 80 and 110%. Comparison of the assay performance on the two qPCR platforms; Rotor-Gene Q and BioMark, revealed only a minor difference in the dynamic range and efficiency for all the assays. For a majority of the qPCRs, the dynamic range was one-two log_10_ higher on the Rotor-Gene Q platform compared to the BioMark. This might be a result of the considerable lower reaction volume in the 48.48DA (< 10 nL) compared to the tubes (25 μl) of the Rotor-Gene Q. No cross reactions were observed for any of the assays on the swIAV 48.48DA, which testifies a high specificity. To test the specificity in more detail, virus isolates and field samples, which have previously been subtyped by sequencing or multiplex RT-qPCR, were tested on the swIAV 48.48DA. Again no cross reactions were observed and the three field samples, which failed to provide a signal in the HA or NA analysis in the multiplex RT-qPCR test, were subtyped by the swIAV 48.48DA. This difference can be explained by the ability of the swIAV 48.48DA to subtype weakly positive samples (Cq-value of 30 or above in the M qPCR assay) which cannot be subtyped using the standard multiplex RT-qPCR protocol. The improved sensitivity of the swIAV 48.48DA is related to the 24 pre-amplification cycles used prior to the PCR step.

The heat map generated by the Fluidigm Real-Time PCR Analysis software illustrates the raw Cq-values for each reaction, which makes is feasible to quickly evaluate which subtype the individual samples have (Figure 2). Using the swIAV 48.48DA for the subtyping of swIAVs in surveillance programs, will make the analysis more simple compared to the traditional subtyping methods and it will give a more detailed subtyping of the samples since the internal genes are included in the analysis.

In summary, the use of the swIAV 48.48DA will allow future subtyping of many more influenza virus isolates for the same resources and by that contribute to a more sensitive surveillance program and provide the basis for an improved early detection of new virus re-assortments and variants. The high sensitivity, specificity and robustness of the test system may also provide an opportunity for development of other similar chips i.e., for the surveillance and diagnose of other veterinary pathogens. Work is in progress on the development of a 48.48DA containing all important swine pathogens for the use in future surveillance and diagnostic programs in Danish swine herds.

## Author contributions

LL, JK, CH, KS, and NG contributed to the experimental design of the study. qPCR assays, which have been designed in the present study, have been designed by JK, TH, SB, and NG. PCR analyses were conducted and interpreted by NG. The main manuscript was initially drafted by NG and LL has contributed to the manuscript preparation, while all authors participated in proofreading of the manuscript. All authors read and approved the final manuscript.

### Conflict of interest statement

The authors declare that the research was conducted in the absence of any commercial or financial relationships that could be construed as a potential conflict of interest.

## Footnotes

^1^http://www.fludb.org

## References

[B1] Alexander D. J. Brown I. H. Harris P. A. McCauley J. W. (1998). Multiple genetic reassortment of avian and human influenza A viruses in European pigs, resulting in the emergence of an H1N2 virus of novel genotype. J. Gen. Virol. 79, 2947–2955. 10.1099/0022-1317-79-12-2947 9880008

[B2] Altschul S. F. Gish W. Miller W. Myers E. W. Lipman D. J. (1990). Basic local alignment search tool. J. Mol. Biol. 215, 403–410. 10.1016/S0022-2836(05)80360-2 2231712

[B3] Bi Y. Xie Q. Zhang S. Li Y. Xiao H. Jin T.. (2015). Assessment of the internal genes of influenza A (H7N9) virus contributing to high pathogenicity in mice. J. Virol. 89, 2–13. 10.1128/JVI.02390-14 25320305 PMC4301103

[B4] Bonin E. Quéguiner S. Woudstra C. Gorin S. Barbier N. Harder T. C.. (2018). Molecular subtyping of European swine influenza viruses and scaling to high-throughput analysis. Virol. J. 15:7. 10.1186/s12985-018-0920-z 29316958 PMC5761149

[B5] Breum S. Ø. Hjulsager C. K. Trebbien R. Larsen L. E. (2013). Influenza a virus with a human-like n2 gene is circulating in pigs. Genome Announc. 1:e00712–13. 10.1128/genomeA.00712-13 24051313 PMC3778196

[B6] Castrucci M. R. Donatelli I. Sidoli L. Barigazzi G. Kawaoka Y. Webster R. G. (1993). Genetic reassortment between avian and human influenza A viruses in Italian Pigs. Virology 193, 503–506. 10.1006/viro.1993.1155 8438586

[B7] Cheung T. K. W. Poon L. L. M. (2007). Biology of influenza a virus. Annals N. Y. Acad. Sci. 1102, 1–25. 10.1196/annals.1408.001 17470908

[B8] Henritzi D. Zhao N. Starick E. Simon G. Foni E. Larsen L. E. (2016). Rapid detection and subtyping of European swine influenza viruses in porcine clinical samples by hemagglutinin- and neuraminidase-specific tetra- and triplex real-time RT-PCRs. Influenza Other Respir. Viruses 10, 504–517. 10.1111/irv.12407 27397600 PMC5059951

[B9] Ishii S. Segawa T. Okabe S. (2013). Simultaneous quantification of multiple food- and waterborne pathogens by use of microfluidic quantitative PCR. Appl. Environ. Microbiol. 79, 2891–2898. 10.1128/AEM.00205-13 23435884 PMC3623133

[B10] Kibbe W. A. (2007). OligoCalc: an online oligonucleotide properties calculator. Nucleic Acids Res. 35, W43–46. 10.1093/nar/gkm234 17452344 PMC1933198

[B11] Korenková V. Scott J. Novosadová V. Jindrichová M. Langerová L. Švec D.. (2015). Pre-amplification in the context of high-throughput qPCR gene expression experiment. BMC Mol. Biol. 16:5. 10.1186/s12867-015-0033-9 25888347 PMC4365555

[B12] Krog J. S. Hjulsager C. K. Larsen M. A. Larsen L. E. (2017). Triple-reassortant influenza A virus with H3 of human seasonal origin, NA of swine origin, and internal A(H1N1) pandemic 2009 genes is established in Danish pigs. Influenza Other Respir. Viruses 11, 298–303. 10.1111/irv.12451 28245096 PMC5410721

[B13] Kuntz-Simon G. Madec F. (2009). Genetic and antigenic evolution of swine influenza viruses in Europe and evaluation of their zoonotic potential. Zoonoses Public Health 56, 310–325. 10.1111/j.1863-2378.2009.01236.x 19497089

[B14] Loeffen W. L. A. de Vries R. P. Stockhofe N. van Zoelen-Bos D. Maas R. Koch G.. (2011). Vaccination with a soluble recombinant hemagglutinin trimer protects pigs against a challenge with pandemic (H1N1) 2009 influenza virus. Vaccine 29, 1545–1550. 10.1016/j.vaccine.2010.12.096 21219983

[B15] Michelet L. Delannoy S. Devillers E. Umhang G. Aspan A. Juremalm M.. (2014). High-throughput screening of tick-borne pathogens in Europe. Front. Cell. Infect. Microbiol. 4:103. 10.3389/fcimb.2014.00103 25120960 PMC4114295

[B16] Pensaert M. Ottis K. Vandeputte J. Kaplan M. M. Bachmann P. A. (1981). Evidence for the natural transmission of influenza A virus from wild ducts to swine and its potential importance for man. Bull. World Health Organ. 59, 75–78. 6973418 PMC2396022

[B17] Simon G. Larsen L. E. Dürrwald R. Foni E. Harder T. Van Reeth K.. (2014). European surveillance network for influenza in pigs: surveillance programs, diagnostic tools and swine influenza virus subtypes identified in 14 European countries from 2010 to 2013. PLoS ONE 9:e115815. 10.1371/journal.pone.0115815 25542013 PMC4277368

[B18] Skovgaard K. Cirera S. Vasby D. Podolska A. Breum S. O. Dürrwald R.. (2013). Expression of innate immune genes, proteins and microRNAs in lung tissue of pigs infected experimentally with influenza virus (H1N2). Innate Immun. 19, 531–544. 10.1177/1753425912473668 23405029

[B19] Spurgeon S. L. Jones R. C. Ramakrishnan R. (2008). High throughput gene expression measurement with real time PCR in a microfluidic dynamic array. PLoS ONE 3:e1662. 10.1371/journal.pone.0001662 18301740 PMC2244704

[B20] Starick E. Lange E. Fereidouni S. Bunzenthal C. Hoveler R. Kuczka A.. (2011). Reassorted pandemic (H1N1) 2009 influenza A virus discovered from pigs in Germany. J. Gen. Virol. 92, 1184–1188. 10.1099/vir.0.028662-0 21307227

[B21] Trebbien R. Bragstad K. Larsen L. E. Nielsen J. Bøtner A. Heegaard P. M.. (2013). Genetic and biological characterisation of an avian-like H1N2 swine influenza virus generated by reassortment of circulating avian-like H1N1 and H3N2 subtypes in Denmark. Virol. J. 10:290. 10.1186/1743-422X-10-290 24047399 PMC3851529

[B22] Watson S. J. Langat P. Reid S. M. Lam T. T.-Y. Cotten M. Kelly M.. (2015). Molecular epidemiology and evolution of influenza viruses circulating within european swine between 2009 and 2013. J. Virol. 89, 840–815. 10.1128/JVI.00840-15 26202246 PMC4577897

[B23] Wu Y. Wu Y. Tefsen B. Shi Y. Gao G. F. (2014). Bat-derived influenza-like viruses H17N10 and H18N11. Trends Microbiol. 22, 183–191. 10.1016/j.tim.2014.01.010 24582528 PMC7127364

