# Supplementary material for: Subtyping of Swine Influenza Viruses Using a High-Throughput Real-Time PCR Platform
Source: Front Cell Infect Microbiol. 2018 May 22;8:165. doi: 10.3389/fcimb.2018.00165 (PMC5972299; doi:10.3389/fcimb.2018.00165)
Supplement: Supplementary file 1 [file Table_1.docx]

Supplementary table 1 GISAID ID number

| Virus isolate | GISAID segment ID number |
| --- | --- |
| A/swine/Denmark/101092-1p1/2013 | PB2: EPI1138374 / PB1: EPI1138375 / PA: EPI1138373 / HA: EPI1138377 / NP: EPI1138370 / NA: EPI1138376 / MP: EPI1138372 / NS: EPI1138371 |
| A/swine/Denmark/101326-1p1/2013 | PB2: EPI1138390 / PB1: EPI1138391 / PA: EPI1138389 / HA: EPI1138393 / NP: EPI1138386 / NA: EPI1138392 / MP: EPI1138388 / NS: EPI1138387 |
| A/swine/Denmark/1013-4p1/2013 | PB2: EPI1138398 / PB1: EPI1138399 / PA: EPI1138397 / HA: EPI1138401 / NP: EPI1138394 / NA: EPI1138400 / MP: EPI1138396 / NS: EPI1138395 |
| A/swine/Denmark/101837-1p1/2013 | PB2: EPI1138422 / PB1: EPI1138423 / PA: EPI1138421 / HA: EPI1138425 / NP: EPI1138418 / NA: EPI1138424 / MP: EPI1138420 / NS: EPI1138419 |
| A/swine/Denmark/10201-2p1/2013 | PB2: EPI1138438 / PB1: EPI1138439 / PA: EPI1138437 / HA: EPI1138441 / NP: EPI1138434 / NA: EPI1138440 / MP: EPI1138436 / NS: EPI1138435 |
| A/swine/Denmark/10365-3p1/2014 | PB2: EPI1138526 / PB1: EPI1138527 / PA: EPI1138525 / HA: EPI1138529 / NP: EPI1138522 / NA: EPI1138528 / MP: EPI1138524 / NS: EPI1138523 |
| A/swine/Denmark/10616-3p1/2014 | PB2: EPI1138534 / PB1: EPI1138535 / PA: EPI1138533 / HA: EPI1138537 / NP: EPI1138530 / NA: EPI1138536 / MP: EPI1138532 / NS: EPI1138531 |
| A/swine/Denmark/10628-3p1/2014 | PB2: EPI1138542 / PB1: EPI1138543 / PA: EPI1138541 / HA: EPI1138545 / NP: EPI1138538 / NA: EPI1138544 / MP: EPI1138540 / NS: EPI1138539 |
| A/swine/Denmark/10781-1p1/2014 | PB2: EPI1138550 / PB1: EPI1138551 / PA: EPI1138549 / HA: EPI1138553 / NP: EPI1138546 / NA: EPI1138552 / MP: EPI1138548 / NS: EPI1138547 |
| A/swine/Denmark/9477-1p1/2014 | PB2: EPI1138574 / PB1: EPI1138575 / PA: EPI1138573 / HA: EPI1138577 / NP: EPI1138570 / NA: EPI1138576 / MP: EPI1138572 / NS: EPI1138571 |
| A/swine/Denmark/10377-1p1/2015 | PB2: EPI1138582 / PB1: EPI1138583 / PA: EPI1138581 / HA: EPI1138585 / NP: EPI1138578 / NA: EPI1138584 / MP: EPI1138580 / NS: EPI1138579 |
| A/swine/Denmark/20566-1p1/2015 | PB2: EPI1138598 / PB1: EPI1138599 / PA: EPI1138597 / HA: EPI1138601 / NP: EPI1138594 / NA: EPI1138600 / MP: EPI1138596 / NS: EPI1138595 |
| A/swine/Denmark/2408-1p1/2015 | PB2: EPI1138622 / PB1: EPI1138623 / PA: EPI1138621 / HA: EPI1138625 / NP: EPI1138618 / NA: EPI1138624 / MP: EPI1138620 / NS: EPI1138619 |
| A/swine/Denmark/3572-1p1/2015 | PB2: EPI1138630 / PB1: EPI1138631 / PA: EPI1138629 / HA: EPI1138633 / NP: EPI1138626 / NA: EPI1138632 / MP: EPI1138628 / NS: EPI1138627 |
| A/swine/Denmark/3627-2p1/2015 | PB2: EPI1138638 / PB1: EPI1138639 / PA: EPI1138637 / HA: EPI1138641 / NP: EPI1138634 / NA: EPI1138640 / MP: EPI1138636 / NS: EPI1138635 |
| A/swine/Denmark/3655-3p1/2015 | PB2: EPI1138646 / PB1: EPI1138647 / PA: EPI1138645 / HA: EPI1138649 / NP: EPI1138642 / NA: EPI1138648 / MP: EPI1138644 / NS: EPI1138643 |
| A/swine/Denmark/4790-1p1/2015 | PB2: EPI1138654 / PB1: EPI1138655 / PA: EPI1138653 / HA: EPI1138657 / NP: EPI1138650 / NA: EPI1138656 / MP: EPI1138652 / NS: EPI1138651 |
| A/swine/Denmark/4804-3p1/2015 | PB2: EPI1138662 / PB1: EPI1138663 / PA: EPI1138661 / HA: EPI1138665 / NP: EPI1138658 / NA: EPI1138664 / MP: EPI1138660 / NS: EPI1138659 |
| A/swine/Denmark/5736-1p1/2015 | PB2: EPI1138670 / PB1: EPI1138671 / PA: EPI1138669 / HA: EPI1138673 / NP: EPI1138666 / NA: EPI1138672 / MP: EPI1138668 / NS: EPI1138667 |
| A/swine/Denmark/5758-1p1/2015 | PB2: EPI1138678 / PB1: EPI1138679 / PA: EPI1138677 / HA: EPI1138681 / NP: EPI1138674 / NA: EPI1138680 / MP: EPI1138676 / NS: EPI1138675 |
| A/swine/Denmark/5775-2p1/2015 | PB2: EPI1138686 / PB1: EPI1138687 / PA: EPI1138685 / HA: EPI1138689 / NP: EPI1138682 / NA: EPI1138688 / MP: EPI1138684 / NS: EPI1138683 |
| A/swine/Denmark/789-2p1/2015 | PB2: EPI1138694 / PB1: EPI1138695 / PA: EPI1138693 / HA: EPI1138697 / NP: EPI1138690 / NA: EPI1138696 / MP: EPI1138692 / NS: EPI1138691 |
| A/swine/Denmark/8913-3p1/2015 | PB2: EPI1138702 / PB1: EPI1138703 / PA: EPI1138701 / HA: EPI1138705 / NP: EPI1138698 / NA: EPI1138704 / MP: EPI1138700 / NS: EPI1138699 |
| A/swine/Denmark/9973-1p1/2015 | PB2: EPI1138710 / PB1: EPI1138711 / PA: EPI1138709 / HA: EPI1138713 / NP: EPI1138706 / NA: EPI1138712 / MP: EPI1138708 / NS: EPI1138707 |
